# Inhibition of Microglial TGFβ Signaling Increases Expression of *Mrc1*

**DOI:** 10.3389/fncel.2020.00066

**Published:** 2020-03-31

**Authors:** Alexander von Ehr, Abdelraheim Attaai, Nicolas Neidert, Phani Sankar Potru, Tamara Ruß, Tanja Zöller, Björn Spittau

**Affiliations:** ^1^Department of Molecular Embryology, Faculty of Medicine, Institute for Anatomy and Cell Biology, University of Freiburg, Freiburg, Germany; ^2^Department of Anatomy and Histology, Faculty of Veterinary Medicine, Assiut University, Assiut, Egypt; ^3^Institute of Anatomy, University of Rostock, Rostock, Germany; ^4^Centre for Translational Neurosciences Rostock, Rostock, Germany

**Keywords:** microglia, *Mrc1*, CD206, TGFβ1, TGFβ signaling

## Abstract

Microglia are constantly surveying their microenvironment and rapidly react to impairments by changing their morphology, migrating toward stimuli and adopting gene expression profiles characterizing their activated state. The increased expression of the M2-like marker *Mannose receptor 1* (*Mrc1*), which is also referred to as CD206, in microglia has been reported after M2-like activation *in vitro* and *in vivo*. *Mrc1* is a 175-kDa transmembrane pattern recognition receptor which binds a variety of carbohydrates and is involved in the pinocytosis and the phagocytosis of immune cells, including microglia, and thought to contribute to a neuroprotective microglial phenotype. Here we analyzed the effects of TGFβ signaling on *Mrc1* expression in microglia *in vivo* and *in vitro*. Using C57BL/6 wild type and *Cx3cr1^*CreERT2*^:R26-YFP:Tgfbr2^*fl/fl*^* mice-derived microglia, we show that the silencing of TGFβ signaling results in the upregulation of *Mrc1*, whereas recombinant TGFβ1 induced the delayed downregulation of *Mrc1*. Furthermore, chromatin immunoprecipitation experiments provided evidence that *Mrc1* is not a direct Smad2/Smad4 target gene in microglia. Altogether our data indicate that the changes in *Mrc1* expression after the activation or the silencing of microglial TGFβ signaling are likely to be mediated by modifications of the secondary intracellular signaling events influenced by TGFβ signaling.

## Introduction

The central nervous system (CNS) is colonized by primitive macrophage precursors from the yolk sac (Ginhoux et al., 2010) during mid- and late-embryonic development, which further give rise to adult microglia involving PU.1- as well as IRF8-dependent signaling pathways (Kierdorf et al., 2013). The CSFR1/IL-34 receptor/ligand pair controls the homing of microglia toward the CNS parenchyma (Ginhoux et al., 2010; Greter et al., 2012), and perinatal microglia maturation is characterized by the establishment of a microglia-specific gene expression pattern involving genes such as *Olfml3*, *Tmem119*, *Hexb*, *Fcrls*, *Tgfbr1*, *P2ry12*, and *Gpr34*, which allows a clear discrimination between the microglia and the other macrophage populations (Gautier et al., 2012; Beutner et al., 2013; Chiu et al., 2013; Hickman et al., 2013). This molecular signature is dependent on the presence of TGFβ1 (Butovsky et al., 2014), which is expressed by neurons located close to the microglia and activates microglial TGFβ signaling at postnatal day 7 (Attaai et al., 2018). Cellular TGFβ1 effects are mediated after binding to TGFβ receptor type 2 (Tgfbr2), followed by the formation of a heteromeric complex including two TGFβ receptor type 2 and two TGFβ receptor type 1 (Tgfbr1) serine/threonine kinases (Yamashita et al., 1994). The proximity of the receptors induces the Tgfbr2-mediated phosphorylation of Tgfbr1, resulting in Tgfbr1-triggered recruitment and the activation of receptor-associated downstream mediators and transcription factors Smad2 and Smad3 (Wrana et al., 1994; Abdollah et al., 1997). Phosphorylated Smad2/Smad3 form a heterotrimeric complex with Smad4, which translocates to the nucleus where Smads interact with the GTCTG/CAGAC palindromic sequences of Smad-binding elements (SBE) located in the gene promoters, to control the expression of TGFβ1 target genes (Massagué and Wotton, 2000).

Microglia represent the CNS-specific immune cell population, are involved in essential physiological CNS functions, and further participate in the development, the progression, and the resolution of pathological conditions (Prinz et al., 2011; Prinz and Priller, 2014). Microglia are constantly surveying their microenvironment and rapidly react to impairments or stimuli by changing their morphology, migrating toward the stimuli and adopting a gene expression profile characterizing their activated state (Butovsky and Weiner, 2018). In analogy to macrophages (Mosser and Edwards, 2008), microglia reactivity has been classified as M1-like and M2-like activation states (Prinz and Priller, 2014). Although these different microglia activation states can be induced *in vitro* by using M1-inducing cytokines such as IFNγ (Zhou et al., 2015) and M2-inducing cytokines such as IL4 (Zhou et al., 2012), these distinct activation patterns do not seem to be applicable *in vivo* (Ransohoff, 2016). However, sophisticated phenotypic microglia characterizations have revealed that distinct temporal and spatial microglia activation states can be observed (Colton and Wilcock, 2010). Homeostatic, developmental white matter-associated, disease-associated, and ageing-associated gene expression patterns can be distinguished from each other, and considerable regional heterogeneity of microglia has been further revealed (Böttcher et al., 2019; Lloyd and Miron, 2019). We have recently reported that microglial TGFβ signaling is essential to maintain a homeostatic microglia phenotype *in vitro* and *in vivo* (Spittau et al., 2013; Zöller et al., 2018b). Using RNAseq-based transcriptomic profiling of *Tgfbr2*-deficient microglia from *Cx3cr1^*CreERT2*^:R26-YFP:Tgfbr2^*fl/fl*^* mice, we observed an upregulation of microglia activation and priming markers indicating an M1-like activation. Interestingly, we further demonstrated the increased expression of the M2-like marker *Mannose receptor 1* (*Mrc1*), which is also referred to as CD206 in microglia with deficient TGFβ signaling (Zöller et al., 2018b). Mrc1 is a 175-kDa transmembrane pattern recognition receptor which binds a variety of carbohydrates and is involved in the pinocytosis and the phagocytosis of immune cells including microglia (Stahl and Ezekowitz, 1998; Martinez-Pomares, 2012). The upregulation of *Mrc1* in microglia has been reported after M2-like activation *in vitro* and *in vivo* (Marzolo et al., 1999; Durafourt et al., 2012; Hu et al., 2012; Kobayashi et al., 2013) and thought to contribute to a neuroprotective microglial phenotype. However, it remains unclear what the functional outcome of *Mrc1* expression in microglia is. In the present study, we analyzed the effects of TGFβ signaling on *Mrc1* expression in microglia. Using C57BL/6 wild type and *Cx3cr1^*CreERT2*^:R26-YFP:Tgfbr2^*fl/fl*^* mice-derived microglia, we demonstrate that silencing of TGFβ signaling results in the strong upregulation of *Mrc1*, whereas treatment with recombinant TGFβ1 leads to a delayed downregulation of *Mrc1*. Furthermore, chromatin immunoprecipitation (ChIP) experiments provided evidence that *Mrc1* is not a direct Smad2/Smad4 target gene in microglia. Altogether our data indicate that changes in *Mrc1* expression after activation or silencing of microglial TGFβ signaling are likely to be mediated by modifications of secondary intracellular signaling events influenced by TGFβ signaling.

## Materials and Methods

### Animals

C57BL/6Rj mice, for generation of primary microglia cultures and acute isolation of microglia at different postnatal ages, were obtained from Janvier (Le Genest-Saint-Isle, France). Cx3cr1^*CreERT2*^:R26-YFP:Tgfbr2^*fl/fl*^ mice, for analysis of TGFβ signaling-deficient microglia, were generated as previously reported (Zöller et al., 2018b). All mice were housed at 22 ± 2°C under a 12-h light/dark cycle with *ad libitum* access to water and food. The animal procedures were performed following the German Federal Animal Welfare Law and the local ethical guidelines of the University of Freiburg. All experimental steps involving mice have been approved by the animal experimentation committee of the University of Freiburg and the Regierungspräsidium Freiburg [G-13/57 (Tgfbr2-MG-KO), X-15/01A (primary microglia)].

### Microglia Cultures

Primary microglia were prepared as previously described (Spittau et al., 2013). Briefly, brains from P0/1 C57BL/6 mice (Janvier) or Cx3cr1^*CreERT2*^:R26-YFP:Tgfbr2^*fl/fl*^ mice were washed with ice-cold Hank’s balanced salt solution (BSS) (PAA, Cölbe, Germany) and the meninges and the vessels were removed. The cleaned brains were collected in ice-cold Hank’s BSS and enzymatically dissociated using 1× Trypsin-EDTA (Thermo Fisher Scientific, Germany) for 10 min at 37°C. An equal amount of ice-cold fetal calf serum (FCS) together with DNase (Roche, Mannheim, Germany) at a final concentration of 0.5 mg/ml was added before dissociation with Pasteur pipettes. The dissociated cells were centrifuged, collected, and resuspended in DMEM/F12 medium containing 10% FCS and 1% penicillin/streptomycin (PAA, Cölbe, Germany). Finally, the suspensions were transferred to poly-D-lysine-coated (Sigma-Aldrich, Schnelldorf, Germany) tissue culture flasks with a density of two to three brains per 75 cm^2^ or one brain per 25-cm^2^ flask. The cultures were incubated in 5% CO_2_/95% humidified air atmosphere at 37°C. At days *in vitro* 2 and 3, the cultures were washed twice with phosphate-buffered saline (PBS), and the fresh culture medium was added. After 7–10 days in culture, the microglia were harvested from adherent astrocytes by shaking at 130 rpm for 1 h. The isolated microglia were seeded into different culture dishes or plates according to the experimental design. Treatment with TGFβ1 (Peprotech, Hamburg, Germany) was performed (2, 6, and 24 h) with a final concentration of 5 ng/ml. For inhibition of microglial TGFβ signaling, a TGFβ receptor type I inhibitor (TβRI) inhibitor (#616454, Calbiochem, Merck, Darmstadt, Germany) at a final concentration of 500 mM was used. Five-day treatments were performed in 75-cm^2^ culture flasks containing mixed glia cultures under serum-containing (10% FCS) conditions. Afterward, microglia were shaken off and used for protein extraction or immunocytochemistry. More than 95% of the isolated cells were microglia as assessed by Iba1 and/or isolectin stainings as previously reported (Spittau et al., 2013). The following densities were used for the *in vitro* experiments involving primary microglia: 700,000 cells/6-cm dish and 70,000 microglia/10-mm glass coverslip. For western blots, microglia were shaken off from 75-cm^2^ flasks after treatment for 5 days, yielding approximately 1 × 10^6^ cells/flask.

### Tgfbr2-Deficient Microglia

*Cx3cr1^*CreERT2*^:R26-YFP:Tgfbr2^*fl/fl*^* mice were used to induce microglia-specific silencing of TGFβ signaling *in vivo* and *in vitro* as recently reported (Zöller et al., 2018b). Briefly, Cre activation was induced *in vivo* by two intraperitoneal injections of 8 mg tamoxifen (T5648, Sigma-Aldrich) solved in 200 μl corn oil (C8267, Sigma-Aldrich) in 6- to 8-week-old mice at two time points 48 h apart. Recombination *in vitro* was induced after treatment of the 25 cm^2^-culture flasks (individual brains) with 4-hydroxytamoxifen (H7904, Sigma-Aldrich) at a concentration of 1 μM for 5 days. Ethanol was used as the solvent control in all *in vitro* recombination experiments.

### BV2 Cell Culture

The murine microglia cell line BV2 was preserved in DMEM/F12 (Thermo Fisher Scientific, Germany) supplemented with 10% heat-inactivated FCS and 1% penicillin/streptomycin (PAA, Cölbe, Germany), and the cells were incubated at 37°C in a 5%-CO_2_-containing 95% humidified air atmosphere. Before the treatments with 5 ng/ml TGFβ1 (Peprotech, Hamburg, Germany) for ChIP experiments, 1 × 10^6^ cells/10 cm dish were washed twice with PBS and kept under serum-free conditions for at least 2 h.

### RNA Isolation and Reverse Transcription

Total RNA was extracted from primary microglia cultures using TRizol Reagent (Invitrogen, Karlsruhe, Germany) according to the manufacturer’s instructions. RNA from acutely isolated microglia was isolated using the RNeasy kit (QIAGEN, Hilden, Germany) according to the manufacturer’s instructions. RNA quality and concentration were determined using the NanoDrop 2000 (Thermo Scientific, Germany). One microgram of total RNA per sample was reverse-transcribed to cDNA using the Protoscript^®^ II First Strand cDNA Synthesis Kit (#E6560S, New England Biolabs, Frankfurt, Germany) according to the manufacturer’s instructions.

### Quantitative RT-PCR

Quantitative RT-PCR (qRT-PCR) was performed using the CFX Connect^TM^ System (Bio-Rad, München, Germany) in combination with the SYBR Green GoTaq^®^ qPCR Kit (A6002, Promega, Madison, WI, United States). Five microliters of cDNA template was used in the 25-μl reaction mixture. The results were analyzed using the CFX Connect^TM^ System (Bio-Rad, München, Germany) Software and the comparative CT method. All data are presented as 2^–ΔΔCT^ for the gene of interest normalized to the housekeeping gene *Gapdh* and presented as fold change relative to the control groups. The following primers have been used throughout this study: Mrc1*for* 5′-TCTTTGCCTTTCCCAGTCTCC-3′, Mrc1*rev* 5′-TGACACCCAGCGGAATTTC-3′ (NM_008625.2), Gapdh*for* 5′-GGCATTGCTCTCAATGACAA-3′, Gapdh*rev* 5′-ATGTAGGCC ATGAGGTCCAC-3′ (NM_001289726), Mrc1-SBE1*for* 5′-CTA GTGCTTGGAAAGCTGATGC-3′, Mrc1-SBE1*rev* 5′-CTCCCC TTATCTCCAACACTACA-3′ (NC_000068.7; Chr. 2; 14226266-14226244), Mrc1-SBE2*for* 5′-AACGGTGGGTCCCTTCT CA-3′, Mrc1-SBE2*rev* 5′-GGCAGGTACACACTCATTTCC-3′ (NC_000068.7; Chr. 2; 14228374-14228354), Mrc1-SBE3*for* 5′-CTTCTGATGCTTTCCAGCGAG-3′ and Mrc1-SBE3*rev* 5′-GTAACCAAACGGAGGCCATT-3′ (NC_000068.7; Chr. 2; 14229128-14229109).

### Immunocytochemistry

Primary mouse microglia were shaken off from mixed glial cultures plated on glass coverslips and incubated for 24 h at 37°C to adhere. Subsequently, the cells were washed with PBS and fixed with 4% paraformaldehyde for 15 min. After washing with PBS (3 × 5 min), the cells were blocked with PBS containing 10% normal goat serum and 0.1% Triton-X 100 (Carl Roth, Karlsruhe, Germany) for 1 h. The microglia were incubated with anti-Mrc1 (sc-58987, Santa Cruz Biotechnology Inc.) at 4°C overnight. After washing three times with PBS, the cells were incubated with Alexa Fluor-568-conjugated secondary antibodies (1:200, Cell Signaling Technology) for 2 h. fluorescein isothiocyanate-coupled tomatolectin (Sigma-Aldrich, Schnelldorf, Germany) was used as a microglial marker, and the nuclei were counterstained with 4′-6′-diamidino-2-phenylindole (Roche, Basel, Switzerland). After final washing (3×), the coverslips were mounted on objective slides using Fluoromount G mounting medium (SouthernBiotech). Fluorescence images were captured using the AxioPlan-2 microscope (Zeiss, Oberkochen, Germany). The fluorescence intensities of *Mrc1* after immunocytochemistry were analyzed using the intensity measurement function of the ImageJ software (National Institutes of Health, Bethesda, MD, United States).

### Protein Isolation and Immunoblotting

Proteins were isolated from primary microglia using RIPA Buffer (Cell Signaling Technology) and the concentrations were measured using Pierce^TM^ BCA Protein Assay Kit (Thermo Fischer Scientific) according to the manufacturer’s instructions. Lysates (10 μg total protein/lane) were loaded on Mini-PROTEAN Precast gels (Bio-Rad, München, Germany) for electrophoresis (∼90 min at 80 V). The proteins were blotted for 10 min onto a polyvinylidene fluoride membrane using the Trans-Blot^®^ Turbo^TM^ RTA Midi PVDF Transfer Kit for the Trans-Blot^®^ Turbo^TM^ Transfer System. The membranes were washed with Tris-buffered saline and blocked with 5% bovine serum albumin (Carl Roth, Karlsruhe, Germany) in TBST for 2 h at room temperature. Subsequently, the membranes were incubated with primary antibody anti-Mrc1 [R&D, Wiesbaden, Germany (FAB2535C), 1:200] or anti-GAPDH [Cell Signaling Technology (#2118), 1:1,000] at 4°C overnight. Finally, the membranes were washed with TBST and incubated with horseraddish peroxidase (HRP)-conjugated donkey anti-goat antibody (Abcam, 1:3,000) and/or HRP-conjugated goat anti-rabbit antibody (Cell Signaling, 1:10,000) for 2 h. The labeled proteins were detected using SignalFire^TM^ ECL Reagent (Cell Signaling, #6883). All blots were captured using the ImageQuant LAS 4000 (GE Healthcare Life Sciences). Densitometric analysis of protein bands were performed using ImageJ software (National Institutes of Health, Bethesda, MD, United States).

### Microglia Isolation and Flow Cytometry

Deeply anesthetized (i.p., injections of ketamin/rompun) C57BL/6 mice were transcardially perfused with ice-cold PBS. After brain dissection and removal of meninges on absorbent paper, the brains were collected in cold buffer (1× Hank’s BSS, 1% BSA, and 1 mM EDTA), homogenized using a glass homogenizer and filtered through a 75-μm cell strainer (Falcon). The cells were centrifuged for 12 min at 300 × *g* and 10°C, and the pellet was resuspended in 5 ml 37% Percoll (P1644, Sigma-Aldrich) in PBS, underlaid with 4 ml 70% Percoll and overlayed with 4 ml 30% Percoll in a 15-ml tube. Percoll gradients were centrifuged for 40 min at 600 × *g* and 10°C without breaks. Finally, the microglia cell layer was collected from the 70 and 37% Percoll interface and transferred to PBS containing 1% FCS and centrifuged for 5 min at 200 *g* and 4°C. Microglia were stained with primary antibodies directed against F4/80 (5 μl, MCA497A488, AbD Serotech) and CD206 (5 μl, FAB2535C, R&D Systems) at 4°C for 15 min. Fc receptor blocking using TrueStain fcX (101319, Biolegend) was used to avoid unspecific antibody binding. The cells were washed and analyzed using the BD Accuri C6 flow cytometer (BD Biosciences).

### Chromatin Immunoprecipitation

ChIP was performed as described by Attaai et al. (2018). The SimpleChIP Enzymatic Chromatin IP kit (Cell Signaling Technology, #9003) was used according to the manufacturer’s instructions. Moreover, 1 × 10^6^ BV2 cells/10 cm cell culture dish were treated with TGFβ1 (5 ng/ml) for 2 h, and proteins were cross-linked with 1% formaldehyde for 10 min. Chromatin digestion was performed with 0.25 μl Micrococcal Nuclease (#10011, Cell Signaling Technology) for 20 min at 37°C. The nuclei were lysed with three sets of 20-s pulses using a Bioruptor sonicator (Diagenode, Liège, Belgium). Normal rabbit IgG (#2729, Cell Signaling Technology) as a negative control, anti-Histone H3 (#4620, Cell Signaling Technology) as a positive control as well as anti-Smad2 (#5339, Cell Signaling Technology) and anti-Smad4 (#38454, Cell Signaling Technology) were used for immunoprecipitation. Chromatin was incubated overnight at 4°C and, after incubating with protein G magnetic beads for 2 h and washing in magnetic separation racks (Cell Signaling, #7017), elution of chromatin was achieved. Cross-linking was reversed using 2 μl Proteinase K (Cell Signaling, #10012) for 2 h at 65°C. Finally, DNA purification in spin columns was performed, and promoter fragments were amplified using qPCR. Data are expressed as 2^–ΔCT^ for the SBE of interest normalized to the rabbit IgG isotype control.

### Statistics

All data are given as means ± SEM. Two-group analysis was performed using Students *t*-test. Multiple group analysis was performed using one-way ANOVA followed by Tukey’s multiple-comparison test. All statistical analyses were performed using GraphPad Prism 8 (GraphPad Software Inc.) and *P*-values <0.05 were considered as being statistically significant.

## Results

### Number of Mrc1^+^ Microglia Decreases During Postnatal Central Nervous System Maturation

**FIGURE 1:**
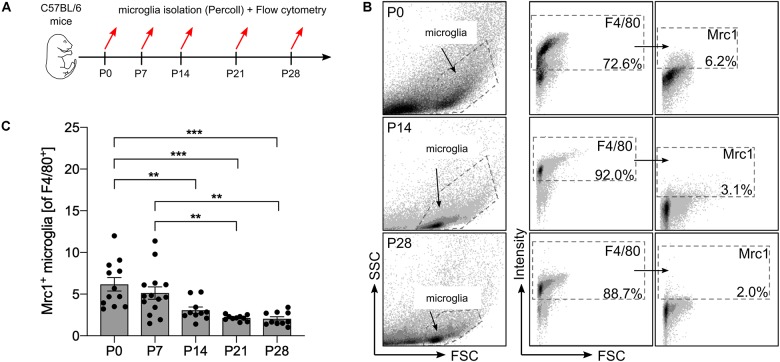
Analysis of postnatal Mrc1 expression in microglia *in vivo*. **(A)** Workflow scheme depicting the time points for acute microglia isolation and flow cytometry. **(B)** Gating strategy and representative dot plots of F4/80^+^ and Mrc1^+^ microglia from P0, P14, and P28 brains. **(C)** Quantification of Mrc1^+^ microglia at the analyzed postnatal developmental stages. Data are given as percentages of F4/80^+^ microglia ± SEM [*n* = 12 (P0), *n* = 14 (P7), *n* = 10 (P14), *n* = 10 (P21), and *n* = 10 (P28)]. *P*-values derived from one-way ANOVA followed by Tukey’s multiple-comparison test are **p < 0.01 and ***p < 0.001.

Using transcriptomic profiling of postnatal microglia, we have recently observed a decreased expression of *Mrc1* from P0 to P28 (Attaai et al., 2018). To validate these observations on functional protein levels, flow cytometry was used to detect the numbers of Mrc1^+^ microglia at different postnatal stages. As shown in Figure 1A, microglia were acutely isolated from mice at postnatal day (P) 0, 7, 14, 21, and 28 using a Percoll gradient and subsequently stained with F4/80 and *Mrc1* for flow cytometry analysis. As depicted in Figures 1B,C, we observed that 6.2% (±0.8%) of all F4/80^+^ microglia were positive for *Mrc1* at P0 and that the numbers of Mrc1^+^ microglia were similar at P7 (5.12 ± 0.74%). Interestingly, a significant decrease in Mrc1^+^ microglia was detected at P14 (3.1 ± 0.39%), P21 (2.14 ± 0.11%), and P28 (2.04 ± 0.25%) compared to the number of Mrc1^+^ cells at P0. Altogether these data indicate that the event which induces the downregulation of *Mrc1* expression in microglia *in vivo* takes place between P7 and P14. It is noteworthy that we have recently determined P7 as the postnatal time point with active microglial TGFβ signaling *in vivo* (Attaai et al., 2018), and thus *Mrc1* downregulation might be the result of Smad2/Smad4-mediated transcriptional regulation.

### TGFβ1 Downregulates the Expression of Mrc1

**FIGURE 2:**
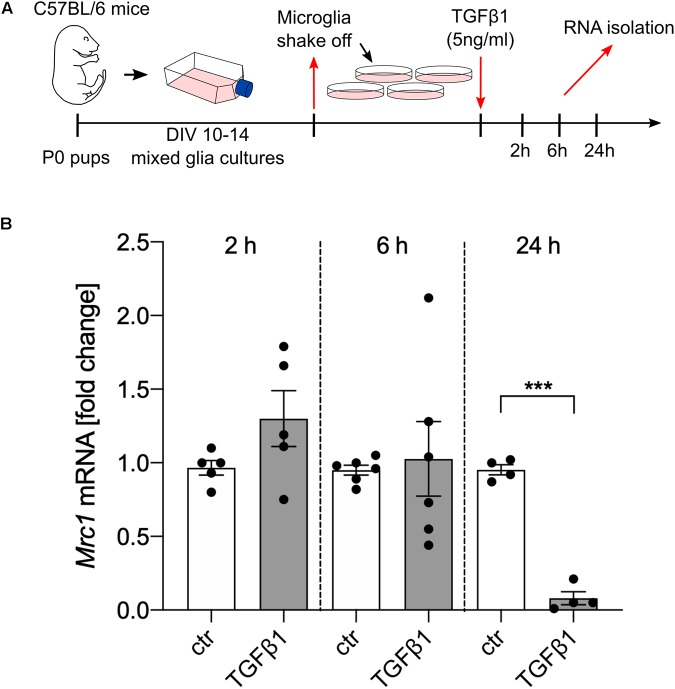
TGFβ1 reduces the expression of *Mrc1* in microglia. **(A)** Scheme displaying the experimental design to analyze the TGFβ1-regulated expression of *Mrc1* in the primary microglia. **(B)** Reduced expression of *Mrc1* in the primary microglia treated with TGFβ1 (5 ng/ml) for 24 h. Treatment for 2 or 6 h did not result in changes in *Mrc1* expression. Data are presented as means ± SEM from five (2 h), six (6 h), and four (24 h) independent experiments. *P*-value derived from Student’s *t*-test is ***p < 0.001.

In order to address whether TGFβ1 is directly able to regulate the transcription of *Mrc1*, primary microglia cultures from C57BL/6 mice were treated with recombinant human TGFβ1 (5 ng/ml) for the indicated time points (Figure 2A). Whereas treatment with TGFβ1 for 2 and 6 h did not result in significant changes of *Mrc1* transcription, a significant downregulation of *Mrc1* was observed after treatment with TGFβ1 for 24 h. These data demonstrate that TGFβ1 induces a robust downregulation of *Mrc1* expression in primary microglia. Moreover, the late time point of TGFβ1-induced *Mrc1* transcriptional inhibition indicates that the *Mrc1* promoter sequences might not be directly regulated by the downstream mediators of TGFβ signaling.

### Inhibition of Microglial TGFβ Signaling Results in Upregulation of Mrc1 Expression

**FIGURE 3:**
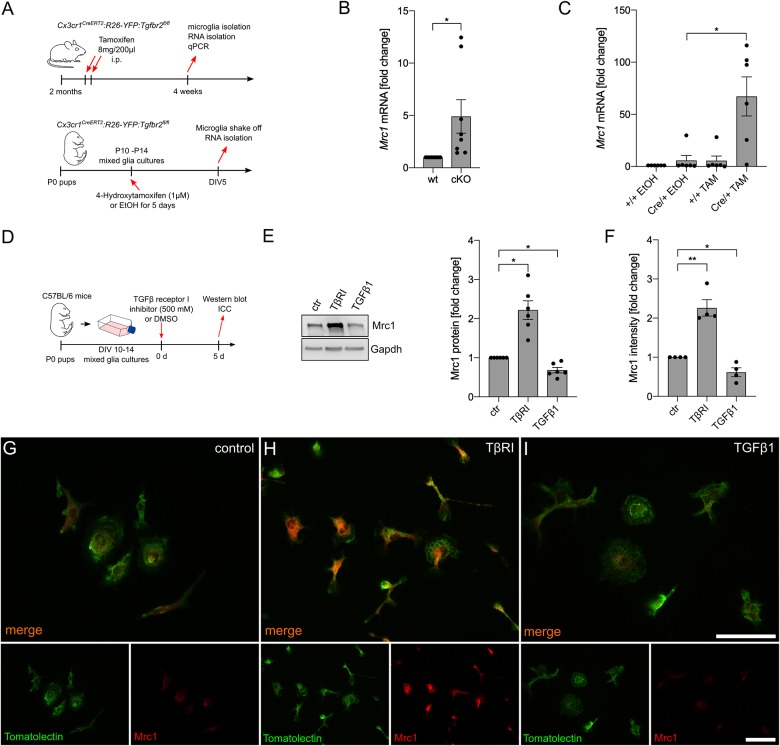
Inhibition of microglial TGFβ signaling results in the upregulation of Mrc1. **(A)** Schemes illustrating the workflow of tamoxifen-induced recombination and microglia isolation from adult *Cx3cr1^*CreERT2*^:R26-YFP:Tgfbr2^*fl/fl*^* mice as well as the tamoxifen-induced recombination and analysis of postnatal microglia *in vitro* isolated from P0 *Cx3cr1^*CreERT2*^:R26-YFP:Tgfbr2^*fl/fl*^* mice. **(B)** Expression of *Mrc1* in adult microglia with intact (wt) and disrupted TGFβ signaling (cKO). **(C)** Cre/+ microglia showed a significantly increased expression of *Mrc1* after tamoxifen-induced recombination *in vitro*. **(D)** Scheme depicting TGFβ receptor type I inhibitor and TGFβ1 treatment for the evaluation of *Mrc1* proteins *in vitro*. **(E)** Representative western blot and quantifications showing the significantly increased protein levels of *Mrc1* after inhibition of microglial TGFβ signaling as well as significant downregulation of *Mrc1* after TGFβ1 treatment for 5 days. **(F)** Quantifications of the *Mrc1* fluorescence intensities after immunocytochemistry reveal a significantly increased intensity after abrogation of TGFβ signaling in the microglia and significant downregulation of *Mrc1* fluorescence intensities after TGFβ1 treatment. Data are given as means ± SEM from at least three independent experiments. *P*-values derived from Student’s *t*-test (**B**) or one-way ANOVA followed by Tukey’s multiple-comparison test **(C)**, **(E)**, **(F)** are *p < 0.05. Immunocytochemistry showing the expression of *Mrc1* in primary microglia after treatment for 5 days. Whereas the control microglia **(G)** and the TGFβ1-treated cells **(I)** show weak immunoreactivity for *Mrc1*, inhibition of TGFβ signaling resulted in increased *Mrc1* staining intensity **(H)**. FITC-coupled tomatolectin was used as a microglia marker. Scale bars represent 50 μm.

In the next step, we aimed to address the effect of silencing TGFβ signaling on microglial *Mrc1* expression *in vivo* and *in vitro*. Therefore, microglia from *Cx3cr1^*CreERT2*^:R26-YFP:Tgfbr2^*fl/fl*^* mice, after tamoxifen-induced recombination *in vivo* and *in vitro*, were used for the analysis of *Mrc1* expression (Figure 3A). As shown in Figure 3B, microglia with conditional knockout of *Tgfbr2* showed a significantly increased expression of Mrc1 *in vivo*. Similar results were observed after tamoxifen-induced knockout of *Tgfbr2* in primary microglia from *Cx3cr1^*CreERT2*^:R26-YFP:Tgfbr2^*fl/fl*^* mice. 4-Hydroxytamoxifen treatment for 5 days resulted in a 67.15-fold (±18.76) increase of *Mrc1* expression in recombined primary microglia (Figure 3C). In order to verify the observed transcriptional changes on protein levels, mixed glial cultures were treated with TβRI, recombinant TGFβ1, or DMSO as the solvent control for 5 days. Afterwards, the microglia were shaken off and used for protein isolation or plated on glass coverslips for immunocytochemistry (Figure 3D). As demonstrated in Figure 3E, immunoblotting revealed a significant increase in *Mrc1* protein levels after TβRI-mediated inhibition of TGFβ signaling and a significant decrease in *Mrc1* protein levels after treatment with recombinant TGFβ1. These observations were confirmed using immunocytochemistry against *Mrc1* and FITC-coupled tomatolectin staining of primary microglia. Whereas weak immunoreactivity for *Mrc1* was detectable in control microglia (Figure 3F), strong cytoplasmic and membrane staining was observed for *Mrc1* after TβRI-induced silencing of TGFβ signaling (Figure 3G). Moreover, immunoreactivity for *Mrc1* was hardly visible after 5 days of incubation with recombinant TGFβ1. Taken together, these data clearly show that silencing of TGFβ signaling either by transgenic (*Tgfbr2* knockout) or pharmacological (TβRI) approaches resulted in the strong upregulation of *Mrc1* in microglia.

### Mrc1 Is Not a Direct TGFβ1 Target Gene

**FIGURE 4:**
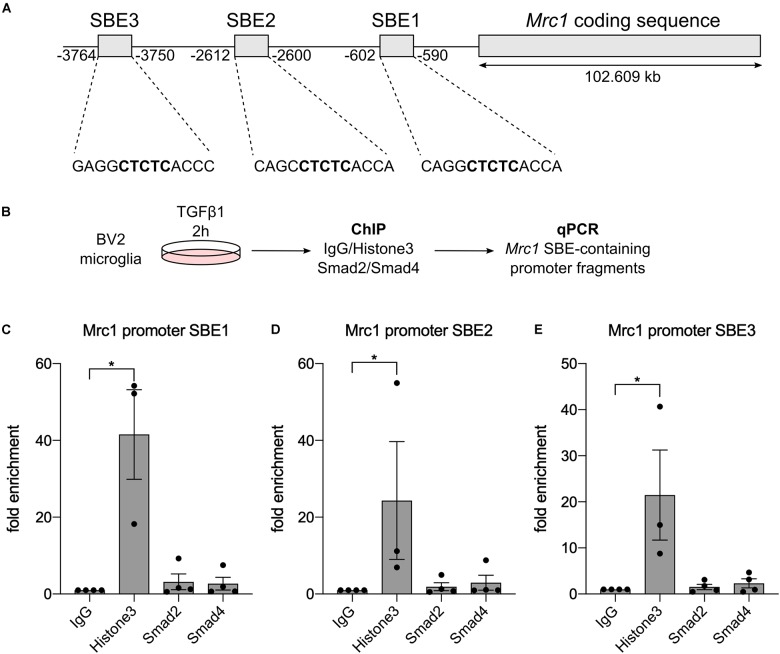
Smad2 and Smad4 are not binding to the *Mrc1* promoter. **(A)** Genomic organization of *Mrc1* depicting the presence of three putative Smad binding elements (SBE) downstream of the transcriptional start site. **(B)** Workflow for chromatin immunoprecipitation procedure. **(C–E)** Quantifications of qPCR-based evaluation of SBE-containing *Mrc1* promoter fragments bound to Histone3, Smad2, and Smad4. Whereas Histone3 significantly enriched the *Mrc1* promoter fragments, neither Smad2 nor Smad4 interacted with putative SBEs. Data are given as means ± SEM from four independent experiments. *P*-values derived from one-way ANOVA followed by Tukey’s multiple-comparison test are *p < 0.05.

To figure out whether Mrc1 is a direct TGFβ1 target gene in microglia, we used ChIP to analyze whether the downstream mediators of TGFβ signaling Smad2 and/or Smad4 interact with Mrc1 promoter elements. In silico analysis of the Mrc1 promoter region revealed the presence of three putative SBE upstream of the transcriptional start site. However, these predicted SBEs contain the palindromic sequence CTCTC, which only partially resembles the GTCTG element for the sequence-specific transcription factors Smad2 and Smad4 (Figure 4A). Since very high cell numbers are needed for ChIP, we used the microglia cell line BV2 after TGFβ1 treatment to validate Smad2/Smad4 binding to the Mrc1 promoter (Figure 4B). As depicted in Figures 4C–E, qPCR-mediated detections of enrichment of SBE-containing promoter fragments could only be observed after pull-down of Histone H3, which served as a positive control during all ChIP experiments performed. Neither pull-down with unspecific control IgG nor precipitation of Smad2 or Smad4 using ChIP-validated antibodies resulted in the enrichment of genomic DNA containing one of the predicted putative SBEs. These data demonstrate that Smad2 and Smad4 are not interacting with the putative SBEs upstream of the Mrc1 transcriptional start site and indicate that Mrc1 is not a direct TGFβ1 target gene in microglia.

## Discussion

In the present study, we have demonstrated that TGFβ1 induces the downregulation of *Mrc1* expression, whereas silencing of TGFβ signaling results in the strong upregulation of *Mrc1* expression in microglia. Moreover, we verified that neither Smad2 nor Smad4 interacts with predicted putative SBE in the upstream regulatory promoter region of *Mrc1*, indicating that TGFβ1-dependent changes in *Mrc1* expression are likely to be mediated by secondary intracellular events triggered by TGFβ1–Smad signaling.

Transcriptional upregulation of *Mrc1* has been shown in mouse macrophages (Martinez-Pomares et al., 2003), and several independent reports have further demonstrated the increased microglial expression of *Mrc1 in vitro* and *in vivo* to be associated with an M2-like microglial activation phenotype. This activation phenotype is commonly induced by IL4 or IL13 (Durafourt et al., 2012; Hu et al., 2012; Kobayashi et al., 2013). IL4, as well as IL13 signaling, is mediated by the receptor-induced activation of tyrosine kinases of the Janus family (Jak), resulting in recruitment, phosphorylation, and dimerization of Stat6 transcription factors which finally mediate the transcriptional control of IL4 and IL13 target genes (Parulekar et al., 2018). Interestingly, the *in silico* promoter analysis for putative SBEs has further revealed the presence of several putative Stat3/Stat5 binding elements (data not shown) which might be involved in the transcriptional upregulation of *Mrc1* after silencing of TGFβ1Smad signaling. Stat3/Stat5 binding sites referred to as STAT-binding elements are usually located in enhancer/promoter regions and first introns of target genes and characterized by clusters of conserved binding motifs with an interferon gamma-activated site-like core sequence (TTCT/CNA/GGAA) (Hutchins et al., 2013; Wingelhofer et al., 2018). It is noteworthy that the activation of JNK/Stat3 signaling has been demonstrated during scar formation as induced by aspirin and was associated with the upregulation of *Mrc1* (Wang et al., 2019). Furthermore, an increased expression of *Mrc1* in macrophages was accompanied by enhanced Jak2–Stat3 pathway activation (Huang et al., 2019), indicating that Stat3 is a potential positive regulator of *Mrc1* expression. The activation of Stat3 has been described to negatively regulate IFN type I responses induced by Stat1 and Stat2 signaling (Wang et al., 2011). IFN-induced Stat3 activation has been reported to inhibit the expression of inflammatory factors *Cxcl9* and *Cxcl10* and further fostering the development of antiviral responses (Ho and Ivashkiv, 2006). We have recently demonstrated that TGFβ1 can reduce Stat1 signaling in primary microglia (Zhou et al., 2015), and it is possible that the silencing of TGFβ signaling results in the over-activation of Stat1 responses which are compensated by increased Stat3 activation in microglia. Interestingly, the inhibition of TGFβ signaling in microglia resulted in the increased secretion of CCL2 and CXCL10, and treatment with recombinant TGFβ1 led to a strong downregulation of Cxcl9, Ccl2, and Cxcl10 (Zöller et al., 2018b). It is noteworthy that it has recently been demonstrated that the CCL2–CCR2 axis is triggering the activation of microglial Stat3 signaling in epileptic mice (Tian et al., 2017). According to the abovementioned findings, *Mrc1* upregulation after inhibition of TGFβ signaling *in vitro* and *in vivo* might be mediated by increased CCL2 release and CCL2–CCR2-driven Stat3 activation in microglia. However, the underlying moelcular signaling events mediating the regulation of microglial *Mrc1* expression need to be further analyzed.

The functional features of Mrc1 involve the binding of glycans and glycoproteins with various configurations of mannose, fucose, and N-acetylglucosamine (Ezekowitz et al., 1991; Kruskal et al., 1992) as well as molecules with the sulfated carbohydrate structure *SO4*-*4*-GalNAcβ1,4GlcNAcβ1,2Manα (Fiete et al., 1998). These capacities allow the interaction of *Mrc1* with glycosylated lysosomal hydrolases (Young et al., 1991), neutrophil granulocyte-derived myeloperoxidase (Shepherd and Hoidal, 1990), and the tissue plasminogen activator (Noorman et al., 1995), suggesting a crucial role of *Mrc1* during the resolution of inflammatory responses. Moreover, *Mrc1* can further interact with the complex surface polysaccharides of microorganisms including bacteria (Pacheco-Soares et al., 1992; Schlesinger, 1993) and viruses (Reading et al., 2000; Lai et al., 2009), thus resulting in their phagocytosis. The ability of *Mrc1* to bind to apoptotic cells and trigger their phagocytic engulfment might be one of the most important functions of microglial *Mrc1* in the CNS (Martinez-Pomares, 2012). This might explain why we have observed a higher microglial *Mrc1* expression at early postnatal time points which are associated with developmental ontogenetic cell death. Interestingly, we have previously shown that aged cortical microglia also express higher levels of *Mrc1* (Zöller et al., 2018a), which might also be part of a microglial response on programmed cell death during aging (Tower, 2015). Human cortical microglia have been demonstrated to express higher levels of *Mrc1* compared to other brain regions (Böttcher et al., 2019). However, it remains unclear what microglia functions *Mrc1* is mediating in the CNS. Recent reports have described a role for *Mrc1* during IL4-induced inhibition of neuroinflammation (Casella et al., 2016) and *Mrc1* upregulation during endotoxin tolerance of BV2 cells after lipopolysaccharide preconditioning (Qin et al., 2016). Taken together, the anti-inflammatory properties have been described for *Mrc1* and the upregulation of microglial *Mrc1* after inhibition of TGFβ signaling might be the result of a compensatory mechanism to prevent the over-activation of the microglia. Our data demonstrate that Smad2/Smad4 are not directly regulating the microglial expression of *Mrc1* and suggest that secondary signaling events guide the transcriptional control of *Mrc1*. Finally, further studies are necessary to elucidate the functional features of microglial *Mrc1* expression under physiological and pathological conditions.

## Data Availability Statement

All datasets generated for this study are included in the article/supplementary material.

## Ethics Statement

The animal study was reviewed and approved by Animal experimentation committee of the University of Freiburg and the Regierungspräsidium Freiburg (G-13/57, X-15/01A).

## Author Contributions

BS conceived and designed the study. AE, AA, NN, PP, TR, and TZ performed the experiments. AE and BS wrote the manuscript.

## Conflict of Interest

The authors declare that the research was conducted in the absence of any commercial or financial relationships that could be construed as a potential conflict of interest.
